# Time to Renitrogenation After Maximal Denitrogenation in Healthy Volunteers in the Supine and Sitting Positions

**DOI:** 10.5811/westjem.2022.5.55378

**Published:** 2022-11-01

**Authors:** Jason R. West, Rykiel Levine, Jason Raggi, Du-Thuyen Nguyen, Matthew Oliver, Nicholas D. Caputo, John C. Sakles

**Affiliations:** * NYC Health + Hospitals | Lincoln, Department of Emergency Medicine, Bronx, New York; † Royal Prince Alfred Hospital, Sydney, Department of Emergency Medicine, New South Wales, Australia; ‡ RPA Green Light Institute for Emergency Care, Sydney, New South Wales, Australia; § University of Arizona College of Medicine, Department of Emergency Medicine, Tucson, Arizona

## Abstract

**Introduction:**

Prior to intubation, preoxygenation is performed to denitrogenate the lungs and create an oxygen reservoir. After oxygen is removed, it is unclear whether renitrogenation after preoxygenation occurs faster in the supine vs the sitting position.

**Methods:**

We enrolled 80 healthy volunteers who underwent two preoxygenation and loss of preoxygenation procedures (one while supine and one while sitting) via bag-valve-mask ventilation with spontaneous breathing. End-tidal oxygen (ETO_2_) measurements were recorded as fraction of expired oxygen prior to preoxygenation, at the time of adequate preoxygenation (ETO_2_ >85%), and then every five seconds after the oxygen was removed until the ETO_2_ values reached their recorded baseline.

**Results:**

The mean ETO_2_ at completion of preoxygenation was 86% (95% confidence interval 85–88%). Volunteers in both the supine and upright position lost >50% of their denitrogenation in less than 60 seconds. Within 25 seconds, all subjects had an ETO_2_ of <70%. Complete renitrogenation, defined as return to baseline ETO_2_, occurred in less than 160 seconds for all volunteers.

**Conclusion:**

Preoxygenation loss, or renitrogenation, occurred rapidly after oxygen removal and was not different in the supine and sitting positions. After maximal denitrogenation in healthy volunteers, renitrogenation occurred rapidly after oxygen removal and was not different in the supine and sitting positions.

## INTRODUCTION

Prior to emergent tracheal intubation, preoxygenation is performed to maintain tissue oxygenation throughout the period when a patient may be apneic.^1^ Rapid sequence intubation (RSI) is the most common medication sequence to facilitate emergent intubation and involves the use of an induction agent and a neuromuscular blocker.^2^ The main purpose of preoxygenation is to maintain tissue oxygenation throughout the apneic period during laryngoscopy as oxygen consumption persists despite the lack of oxygen flow.^1^,^3^ If a patient is unable to tolerate preoxygenation, delayed sequence intubation (DSI) can be performed by administering the induction agent first to facilitate preoxygenation prior to administering the neuromuscular blocker.^4^ Without adequate preoxygenation, a patient undergoing RSI may desaturate quickly because denitrogenating the functional residual capacity (FRC) and formation of an alveolar oxygen reserve did not occur.^5^–^11^ Preoxygenation is recommended to be measured using end-tidal oxygen (ETO_2_), where available, with an ETO_2_ concentration of >85% considered to be adequate.^12^

Preoxygenation efforts should continue to the onset of apnea, but sometimes the oxygen source is removed prior to the onset of apnea resulting in a potential loss of adequate preoxygenation. Spontaneous respiration of room air after the preoxygenation delivery device is removed will result in loss of preoxygenation and reduced safe apnea time.^13^ Most emergency department intubations occur in the supine position.^14^ However, some have advocated for emergent tracheal intubation to occur in an inclined or head-of-bed elevation position, which may improve laryngeal view,^15^ increase first-pass success rate,^16^ and decrease peri-intubation complications.^17^ Furthermore, head-of-bed elevation likely improves preoxygenation and extends the safe apneic period.^18^–^21^

Although preoxygenation in the head-of-bed elevated position may help alveolar oxygenation compared to the supine position, it is unknown whether the loss of adequate preoxygenation is affected by patient positioning. We sought to investigate whether the upright position would result in reduced preoxygenation loss compared to the supine position when oxygen delivery was removed. The purpose of this study was to determine the rate of preoxygenation loss in healthy individuals in both the supine and upright positions.

## METHODS

### Setting

We conducted a prospective, cohort crossover study of healthy volunteers at two urban, academic teaching hospitals near sea level in New York City, NY, and Sydney, Australia. The volunteers were resident physicians recruited when they were available, and no compensation for enrollment was given. This study was approved by the Institutional Review Board and the Ethics Board of the New York City and Sydney hospitals, respectively. We recruited healthy volunteers who consented to participation.

### Measurements

Demographic details such as age, weight, height, and smoking status were taken for each volunteer. All subjects were preoxygenated via bag-valve-mask ventilation in the supine position after being instructed to breath normally until their ETO_2_ was >85% or until their ETO_2_ had plateaued on the maximum oxygen flow rate, which was 15 liters per minute (L/min) at the Sydney site and 50 L/min at the NYC site. End-tidal oxygen was measured as a fraction of expired oxygen (FeO_2_) using a Philips G5 gas analyzer (Philips Healthcare, Cambridge, MA) at the NYC site and a Philips G7 gas analyzer at the Sydney site. While remaining in the supine position, the oxygen supply was removed after optimal preoxygenation and ETO_2_ levels were recorded in five-second intervals until they reached baseline values consistent with continuously breathing room air. This process was repeated for each patient in the upright, sitting position.

### Statistical Analysis

As this was a pilot study of volunteers describing the rate of renitrogenation, and in the setting of the lack of prior data, an a priori sample size and power analysis was not performed. We calculated mean measurements with 95% confidence intervals (CI). We plotted the ETO_2_ measurements over time after maximal preoxygenation and removal of the oxygenation source to measure renitrogenation and plotted the 95% confidence interval (CI) of the mean ETO_2_ values in five-minute intervals. We performed statistical analysis using XLSTAT (Addinsoft, Inc, New York, NY). Data were analyzed using repeated measures ANOVA, using the position (supine vs seated) and time (seconds) as factors to evaluate oxygen levels over time in the two positions. Statistical significance was accepted at *P* <0.05. Statistical analysis for the repeated measures ANOVA was performed via SPSS v 26 (IBM Corp, Armonk, NY).

## RESULTS

We enrolled 80 volunteers. The mean age was 29 (95% CI 26–31), and the mean body mass index was 24 (95% CI 23–25). All volunteers were non-smokers. The mean baseline ETO_2_ was 16% (95% CI 16–17%). Only 12 (15%) volunteers required more than three minutes to achieve an ETO_2_ >85%, and the remainder achieved this goal in less than three minutes. The mean ETO_2_ at completion of the preoxygenation process was 86% (95% CI 85–88%). Loss of preoxygenation was detectable at five seconds after oxygen delivery device removal. Within 25 seconds all ETO_2_ values were less than 70% in both the supine and upright positions. Preoxygenation loss of 50% or greater occurred in less than 60 seconds (Figure). Complete renitrogenation, defined as return to baseline ETO_2_, occurred in less than 160 seconds for all volunteers. The repeated measures ANOVA analysis indicated there was no difference in ETO_2_ over time between the seated and supine position (*P* = 0.48).

## DISCUSSION

In this group of healthy volunteers, renitrogenation occurred rapidly after maximal preoxygenation. Within just 25 seconds, all ETO_2_ values were less than 70% in both the supine and upright positions. End-tidal oxygen values <70% have been cited as inadequate in related literature.^13^ Prior to laryngoscopy, if room air is being entrained into the FRC of an ill patient, adequate oxygen reserve may be lost. This may have major implications for patients where oxygen reserve is decreased before the apneic period or in cases where the mask seal is broken, potentially leading to hypoxia and adverse patient outcomes. Our results are consistent with a similar study by Mosier et al^13^ who concluded that loss of preoxygenation in healthy patients occurred rapidly if oxygen sources were removed. Our study continued ETO_2_ measurements until they reached baseline values, and this allowed us to demonstrate renitrogenation, or deoxygenation, curves in healthy individuals after maximal preoxygenation. Within 160 seconds of breathing room air after maximal preoxygenation, all volunteers returned to their baseline ETO_2_ measurement.

Although preoxygenation has been previously studied in healthy volunteers while comparing sitting and supine positions, no studies have investigated the rate of renitrogenation in both positions.^22^ In this study we did not find that the sitting position reduced preoxygenation loss compared to the supine position, using repeated measures ANOVA analysis. Previous studies have suggested that placing the patient in a head-of-bed elevated position may improve preoxygenation.^18^–^21^ Compared to a supine position, a sitting position has generally been shown to increase the forced vital capacity (FVC),^23^–^25^ especially among those with heart failure,^25^,^26^ and increase the FRC.^25^,^27^,^28^

The increase in FRC is likely due to diaphragmatic descent and reduced pulmonary blood volume. It is possible that increased FRC was obtained by our patients in the sitting position to some degree, but did not manifest as retained preoxygenation, especially considering that total lung and residual volumes are unlikely to be affected by patient positioning.^27^,^29^ Additionally, diffusing capacity is decreased in the sitting position compared to the supine position^30^; and this may reflect an increase in pulmonary capillary blood volume in the supine position.^31^ Furthermore, it is possible that position-related changes regarding airway closure, such as a reduced closing capacity in the supine position,^32^ were blunted because this effect is thought to increase with advanced age.^33^

Our study suggests that neither the supine nor the upright position hold an advantage over the other in terms of maintenance of denitrogenation during the hypoventilatory period prior to the onset of apnea or during preoxygenation in the presence of a mask leak. Our results are consistent with those of Mosier et al^13^ and highly emphasize that preoxygenation devices should be left in place after the RSI drugs are administered and continue to deliver oxygen until the patient is determined to be ready for laryngoscopy and subsequent blade entry into the oropharynx. Our results suggest that when a patient becomes uncooperative with preoxygenation (removing a preoxygenation device or introducing a mask leak) prior to emergent tracheal intubation via RSI, DSI should be considered to avoid preoxygenation loss that may occur seconds before the RSI drugs would be administered.

Perhaps our renitrogenation curve data is most applicable to patients receiving drug-assisted intubation, where a neuromuscular blocking drug is not administered^34^ or when intubating patients for elective procedures. Our results also highlight that rapid loss of preoxygenation adequacy, whether due to mask leak or suboptimal oxygen delivery, can occur prior to laryngoscopy in patients undergoing emergent RSI and that ETO_2_ monitoring could measure this loss in real time.^35^,^36^

## LIMITATIONS

The first limitation to consider is that our study population comprised healthy volunteers who were not likely to have active lung pathology or pulmonary shunting that would be seen in critically ill patients requiring emergent RSI. Secondly, the oxygen demand of our volunteers was likely lower than those requiring intubation for critical illness; therefore, it is likely that our results underestimate the rapid loss of preoxygenation in critically ill patients after oxygen delivery is removed. Third, our volunteers were relatively young and without known lung or cardiac pathology that may have caused our results to be different, since patient positioning from supine to sitting does not have consistent effects on pulmonary function tests between healthy and non-healthy patients.^24^–^26^,^28^ Fourth, including obese subjects instead of healthy subjects could have changed the mean ETO_2_ values observed. Finally, we did not administer RSI medications, which can cause hypoventilation that could slow renitrogenation or affect air entrainment.

## CONCLUSION

In healthy volunteers breathing spontaneously, preoxygenation loss of 50% or greater occurred in less than 60 seconds after the oxygen delivery device was removed, highlighting the importance of a tight mask seal during preoxygenation. Loss of preoxygenation was detectable at five seconds after oxygen delivery device removal; and within 25 seconds, all patients had an ETO_2_ <70%. Preoxygenation loss was not different in the supine and sitting positions. Operators performing intubation in the ED should be cognizant of the rapid loss of preoxygenation and avoid removing the oxygen delivery source until there is complete apnea.

## Figures and Tables

**Figure:**
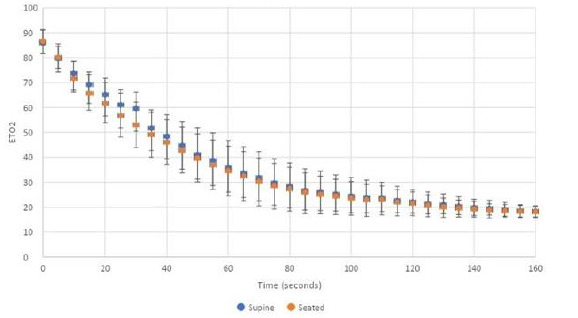
Renitrogenation after maximal preoxygenation in the supine and seated positions.
